# Analyzing the Difficulties of Continuing Physical Activity during the COVID-19 Crisis in France

**DOI:** 10.3390/ijerph19063539

**Published:** 2022-03-16

**Authors:** Coralie Dumoulin, Nathalie Havet, Jean-Yves Lesueur

**Affiliations:** 1 Chaire Prevent’Horizon, Laboratoire Reshape Inserm U 1290, 69373 Lyon, France; 2 Laboratoire de Sciences Actuarielle et Financière (LSAF) EA 2429, Institut de Science Financière et d’Assurances (ISFA), Université Claude Bernard Lyon 1, 69007 Lyon, France; nathalie.havet@univ-lyon1.fr; 3 GATE L-SE UMR 5824, Laboratoire de Sciences Actuarielle et Financière (LSAF) EA 2429, Université de Lyon, 69007 Lyon, France; jean-yves.lesueur@univ-lyon1.fr

**Keywords:** physical activity, sedentary behavior, lockdown, health

## Abstract

Physical activity (PA) and limiting sedentary behavior have been recognized as health-promoting behaviors for many years. Since the COVID-19 pandemic, changes in lifestyle habits have occurred, causing disparities in PA practice. This article aimed to examine the characteristics of French adults who self-reported having difficulties in continuing their exercise practices during the pandemic. Multivariate logistic regressions were used to test whether certain demographic, morphologic, behavioral (sleep, sedentary lifestyle, extent of household chores), and exercise-related variables were significant predictors of experiencing such difficulties, based on data from an online survey of insurance company members. Difficulties in PA practice were found in 57% of the population surveyed. Several factors were identified as predictors of experiencing difficulties, including a high BMI, the type and number of physical activities usually practiced before lockdown, as well as the number of times per week dedicated to PA. For the employed population, specific factors were additionally decisive: sex, time spent in front of screens, and sleeping. Our results will allow public health policy makers and stakeholders in PA and prevention to better target populations in difficulty during periods of disruption, such as that of the pandemic; thus, allowing them to propose structural or organizational solutions for the continuity of PA practice.

## 1. Introduction

Health benefits induced by regular physical activity (PA) are now well documented and their effects demonstrated, regardless of age and sex [1]. For example, regular physical activity, even of moderate intensity, reduces overall mortality, increases life expectancy, and is associated with a greater likelihood of living to an old age in good condition [2,3,4]. It is also one of the factors in the prevention and treatment of major chronic conditions (cancer, cardiovascular diseases, obesity and type 2 diabetes, neurological, rheumatic, and degenerative diseases, etc.) [5,6,7,8,9], and is known to be associated with improved mental health, decreasing the levels of anxiety and depression [10,11]. Therefore, the current recommendation of the World Health Organization (WHO) for healthy adults is to do at least 150 min of moderate aerobic exercise or at least 75 min of vigorous aerobic exercise during the week [12]. This is in addition to muscle-strengthening activities, involving major muscle groups, on two or more days weekly [13]. However, in 2014–2016, only 71% of men and 53% of women were sufficiently active in France, according to these international recommendations [14]. Globally, nearly one adult in four did not reach these minimal recommendations [15,16]. Following these results, the WHO developed a new Global Action Plan to promote PA in 2018–2030 [12].

However, with COVID-19 striking globally, compliance with and commitment to continuing PA seems much more challenging, even for active adults [17]. A number of studies have thus sought to measure the impact of the COVID-19 pandemic on PA participation [18]. The main concern was that lockdowns, partial or total closures of certain recreational venues (restaurants, cinemas, gyms), and other restrictions on certain sports activities due to restrictive health measures significantly decreased individual PA, both regarding frequency and intensity, along with the associated benefits. An additional concern was that the pandemic may have caused psychological disruption that could also affect the sustainability of PA. The crisis context, which is stressful and anxiety-provoking for many people, can have negative impacts on the desire and motivation of exercisers, which can be exacerbated by changes in daily life habits (telecommuting, childcare, or different school restrictions), making it necessary to find a new balance to reconcile professional and domestic activities with PA. Furthermore, some participants may be reluctant to return to sports clubs or to renew their membership for fear of being exposed to the virus and/or of experiencing new closures of sports facilities and suspensions of group activities [19]. Consequently, the decrease in PA levels may be very pronounced in active adults habitually practicing sports [20].

Overall, studies around the world showed a negative impact on PA during COVID-19 health restrictions, although the severity of lockdown has varied from country to country, with some countries limiting the distance that people could travel from their homes, time spent outdoors, and some banning any unnecessary activity [18,21]. Some studies [18] have examined PA changes in children and adolescents, but these populations are not addressed in our study. For adult populations, nearly all of the studies that focused on changes in time spent on PA reported decreases in the amount of PA pre-COVID-19 versus post-COVID-19 lockdown [18,22,23,24,25]. Similarly, studies that measured PA changes as a percentage of the respective adult populations overwhelmingly reported that more than 50% of the examined population decreased PA during lockdown [19,22,26,27,28,29]. For example, in France, where 55 days of lockdown were imposed on the population from 17 March to 11 May 2020, and where the closure of sports facilities continued after this date, more than one in two adults declared having decreased PA during the lockdown periods [22,27]. Thus, in adult populations, PA during lockdown periods decreased compared with pre-lockdown, despite various government organizations and health or exercise practitioners providing guidance on how to stay active during the pandemic and in self-quarantine [30,31].

However, behind these overall figures are exceptions [32,33] and disparities according to certain socio-demographic characteristics (e.g., age, education level, living situation—alone or in urban areas) [23,33,34] or by the activity status of individuals pre-lockdown [25,34,35]. In particular, previous studies stratifying between pre-lockdown PA levels found that people who were more active pre-lockdown were more likely to show larger decreases in PA [23,35]. Given this background and in order to better identify behaviors that are more or less deleterious to the sustainability of PA during a period of disruption, this study aimed to examine characteristics of French adults who self-reported having difficulties staying physically active during the health crisis. Using a sample of adults who were physically active before the lockdown from an online survey of members of a group of French mutual insurance companies heavily involved in health prevention, we tested whether certain demographic, morphologic, behavioral (sleep, sedentary lifestyle, extent of household chores), and exercise-related variables were predictors of experiencing such difficulties. Understanding the difficulties encountered by physically active individuals in their daily practice during lockdown periods is important, not only for associated health outcomes, but also for aiding the development of public health interventions in specific populations should future lockdown periods be enforced, or a similar pandemic scenario occur.

## 2. Materials and Methods

### 2.1. Sample

Participants in the survey had to be over the age of 18 and be insured by a group of French mutual insurance companies that deployed the online survey to their policyholders (i.e., not only individuals in employment, but also unemployed and retired individuals) via several internal communications (emails and a newsletter). Among the respondents to the online survey, only questionnaires with complete answers and individuals who reported practicing PA before the first lockdown period were included in the study. A resulting sample of 460 adults was included in the study.

### 2.2. Data Collection

Participants completed an online survey using online software (Eval&Go) (Evalandgo SAS, Montpellier, France) between 1 September 2020 and 31 December 2020. The survey, developed by Chaire Prevent’Horizon, was composed of three different sets of questions: (1) demographics and morphology, (2) physical and sedentary activity behavior, and (3) domestic and familial activities. Demographic and morphological characteristics included age, sex, residential status (three categories: lives alone, as a couple, with others), and body mass index (BMI). Additionally, occupational status (regrouped into six categories: retired persons, civil servants, intellectual professions/managers, clerks, service workers, other) and annual taxable household income (regrouped into five categories: <EUR 20,000; EUR 20,000–30,000; EUR 30,000–50,000; EUR 50,000 and above; non-responses) were captured.

The evaluation of PA, sedentary behavior, and extent of household chores of the respondents refers mainly to the period outside of lockdown, except for our variable of interest. To cope with the health crisis caused by the COVID-19 global pandemic, 55 days of lockdown were imposed on residents in France from 17 March 2020 to 11 May 2020, and closures of sports facilities (e.g., swimming pools, gyms, etc.) lasted beyond that date. Thus, at the time of the survey, access to these facilities had not yet returned to normal, and the rhythm of life and daily habits of the French residents, including the individual practice of PA, were once again be disrupted by curfews and other restrictive measures for a return to the workplace. Therefore, contrary to previous studies, we did not attempt to compare PA levels “before” and “after” lockdown. We aimed to understand which individual profiles were more likely to be associated with self-reported difficulties in remaining physically active during the health crisis. Consequently, the first question in the survey concerning PA was: “Before the lockdown, did you practice regular physical activity (i.e., one or more times per week)?” We retained in our sample only those who answered affirmatively. Their difficulties in practicing PA during the health crisis were assessed via the following simple yes/no question: “Did/Do the health crisis-related closures of gyms, clubs, and other facilities cause you any problems in your physical activity?” To verify if the answer to that question depended on the nature of the PA usually practiced, all the individuals of the sample were required to provide details on their pre-lockdown PA (frequency, duration, types of activities) by answering the following additional questions: “Before the lockdown, (i) how many times per week did you usually practice physical activity?” (ii) “How much time per week did you usually spend in physical activity?” (iii) “What types of physical activities did you usually participate in?” (For these questions, a list of possible PA activities was provided.) Similarly, participants were asked to report the average time they usually spent per day in front of screens prior to the lockdown, the number of hours they slept per night, and the average number of hours they spent doing household chores per week. The questions were: “Before the lockdown, (i) how much time, on average, did you spend in front of a screen—tablet, computer, phone, etc.—per day (including worktime)?” (ii) “How long did you sleep per night on average?” and, (iii) “How much time did you spend doing housework (cleaning, ironing, cooking, etc.) per week?”

### 2.3. Statistical Analysis

A descriptive approach was used to examine which profiles had difficulties in continuing PA during the health crisis period. The differences in the characteristics of individuals who did and did not experience such difficulties were studied using mean-comparison t-tests for continuous data and exact chi-square tests for categorical data. However, as all individual factors are correlated, the impact of each variable on the probability of experiencing difficulties can only be accurately evaluated on an “all other things being equal” basis by means of multivariate models. Consequently, we used multivariate logistic regressions analysis to study the determinants of the probability that the health-crisis-related closure of gyms, clubs, and other facilities have caused difficulties regarding individual PA practice. The dependent variable was binary, 1 if the participant self-reported such difficulties and 0 if otherwise. Demographic and morphological characteristics, as well as PA, sedentary, and domestic behaviors before the COVID-19 lockdown were retained as covariates in order to identify significant predictors of experiencing such difficulties.

Analyses were conducted, not only on all 460 participants who met the required inclusion criteria, but also on the subsample of 238 participants in employment. Regarding the difficulties encountered, it is conceivable that barriers other than the closure of facilities could have motivated some people to report difficulties in being physically active during the pandemic. For example, a change in work design during the lockdown could be argued as a reason for reduced PA. Consequently, the explanatory factors of experiencing difficulties could differ between the general population (containing retired and unemployed persons) and the employed population. The comparison of both regressions allows us to identify potential discriminant effects of covariates for the employed population that could support the work design change (such as working from a home office) during the lockdown. All analyses were performed using Stata V.16.1 software (StataCorp, College Station, TX, USA) and significance was set at *p* < 0.10.

## 3. Results

### 3.1. Sample Characteristics

Table 1 presents the characteristics of the samples (total and in employment). In our total sample, participants were on average 57 years old (SD = 12.4, min: 21, max: 89) and almost two thirds were women (68%). Most participants lived as a couple (58%) while almost one third lived alone. Forty-seven percent of the individuals were retired, nearly 20% were managers/executives, and 15% were civil servants. Concerning their regular PA, the frequency of practice was around 4 times per week before the health crisis (SD = 1.9, min: 1, max: 10) for a total average weekly duration of 5 h (SD = 3.2, min: 1, max: 15). They practiced on average more than 2 different physical activities (SD = 1.2, min: 1, max: 6). Nevertheless, the most frequent answer in the activities practiced remained walking only (for 10% of the respondents), followed by walking combined with cycling (5.7%), and walking combined with yoga/meditation (4.8%). Participants spent an average of over 5 h per day in front of a screen, slept over 7 h per night, and spent an average of 5.5 h per week doing housework before the lockdown.

Compared to the total sample, the employed population was characterized by a lower average age (48.7 years, SD = 10.2, min: 21, max: 75), higher household income, and significantly higher screen time per day (7.41 versus 5.33), at the expense of a lower frequency and duration of weekly PA.

### 3.2. Mean and Frequencies Comparison Test Results

In our total sample, 57.4% of participants (*n* = 264) reported difficulties in continuing regular PA during the COVID-19 pandemic. Descriptive statistics and non-parametric comparison tests highlighted that individuals experiencing such difficulties exhibited different characteristics than those who did not experience such difficulties (Table 1). For example, more women than men experiences these difficulties (*p* < 0.001). Participants who experienced difficulties were also characterized by a lower PA frequency per week (*p* < 0.001) and a lower average time spent in PA per week (*p* = 0.016) prior to the lockdown period. However, they usually practiced a higher number of different physical activities (2.5 versus 2) and performed more hours of housework per week, on average. In contrast, groups did not differ significantly regarding occupational status and annual household income.

When the sample was restricted to the employed population, the proportion of participants who reported difficulties remained stable (57.6%, *n* = 137) and the same differences were observed overall between participants with and without difficulties (Table 1). There was no significant difference in the average time of PA per week between the two groups (*p* = 0.189). However, among the employed population, there was a significant difference in the daily time spent in front of a screen (*p* = 0.040): individuals who had difficulties in maintaining PA during the pandemic spent more time on screens prior to the lockdown.

### 3.3. Multivariate Analysis Results

Table 2 displays the results of the multivariate logistic regressions for our total sample and the subsample of employed individuals, explaining whether participants experienced PA practice difficulties during the pandemic. Although there are some common explanatory factors for these difficulties in the employed population and the total population, there are some specific predictors associated with each sample.

The common elements are related to morphology and PA parameters. Specifically, regardless of the population considered, the probability of experiencing difficulties in maintaining PA during the lockdown increased significantly with an increase in body mass index (BMI) and the number of different physical activities practiced regularly before the lockdown. Conversely, the higher the frequency of PA per week before lockdown, the fewer difficulties they encountered in maintaining it. In addition, the type of activities practiced before the lockdown emerged as an important determinant. People practicing sports activities mainly outdoors and not requiring the opening of specific sports facilities generally had less difficulty maintaining PA during lockdown. This was particularly the case for those who practiced only cycling, walking, or running, or a combination of these sports (running + walking, walking + cycling, or another combination with cycling).

In contrast, individual and socioeconomic characteristics had different effects according to the considered sample. On the one hand, in the subsample of employed individuals, only the sex variable was statistically significant: all other things being equal, men had less difficulty than women in continuing PA, but this sex effect was not apparent in the total sample. On the other hand, in the total population, income and socio-professional category (to a lesser extent, *p* < 0.10) played a significant role in the likelihood of encountering difficulties in continuing to practice PA. Individuals whose household income was between EUR 30k and 50k (i.e., close to or above the French median income) had more difficulties in continuing to practice PA during the health crisis than those in other categories. In addition, managers and service workers had fewer difficulties than retirees (reference category).

The variables reflecting sedentary behavior and sleep quality were only significant for the employed population. The higher the daily sleep time and the lower the screen time before lockdown, the less difficulty they reported in continuing PA during the health crisis.

## 4. Discussion

Studies conducted during the COVID-19 period indicate, for the vast majority, a decrease in daily PA coupled with an increase in sedentary behavior [18]. However, other studies have also shown the deleterious effects of these trends on the health of individuals, including a deterioration in mental health and a reduction in immune defenses [30]. Therefore, various government organizations and health experts advocated that individuals should remain physically active to prevent future health problems and a subsequent decline in quality of life [36].

Within this context, we were interested in the demographic and pre-pandemic exercise-related characteristics of individuals who reported difficulties in pursuing PA during the health crisis. Gaining knowledge about these factors is of utmost importance. This allows prioritizing and quickly targeting—from the beginning of a pandemic episode (or a period of disruption of habits of any kind), without waiting for the appearance of negative effects or measuring the actual decrease in PA—the populations in greatest need of support and innovative tools (i.e., video-, app-guided, equipment-free, and other home-based digital technologies) that support remote PA. This could, in turn, encourage more people to stay physically active, which might considerably influence their ability to cope with the current pandemic and other outbreaks of infectious disease by reducing stress and anxiety while increasing well-being, and the quality of sleep and life [37].

According to our study, in which 57% of the participants reported difficulties in continuing PA, the pre-pandemic exercise-related characteristics seem to be the most important determinants of the sustainability of PA practice. This finding is consistent with results from other countries [38]. While previous studies have shown that the pre-lockdown activity status of individuals needs to be taken into account to understand the effect of COVID-19 restrictions on PA, by focusing on the three usual dimensions of PA (frequency, duration, intensity) [33,39], our results highlight that the number and the type of physical activities usually practiced have a strong influence. Not surprisingly, outdoor activities, such as walking and running, significantly reduced the probability of experiencing PA practice difficulties during the health crisis and could thus be considered as favorable predictors to the sustainability of the practice. Moreover, the statistics from the 2020 National Barometer of Sports Practices support this view, as they show that these two activities were the most pursued during the lockdown in France [40]. The closure of sports facilities, which has contributed to decreasing the practice of other physical activities, has undoubtedly strongly contributed to these results. However, since our regressions control for the number of different activities usually practiced explicitly specifies some combinations of PA as covariates, we can go a bit further in our analysis. For a given number of PA usually practiced, the effects of the nature of the activities practiced could be determined. Among the individuals who practiced one regular activity before lockdown, those individuals who ran, cycled, or walked experienced fewer difficulties in continuing their activity than those who participated in other activities. However, despite the development of numerous videos, applications, or video courses to encourage individuals to continue PA at home, individuals who participated in muscle strengthening exercises reported as many difficulties in maintaining PA as individuals who practiced sports that are highly dependent on specific infrastructures without real substitutes (e.g., swimming). This result points out the need for further studies on the use and content of digital technologies dedicated to PA and their substitutability/complementarity with indoor activities. For individuals who usually practiced two regular activities before lockdown, only the combinations of running + walking, cycling + walking, and cycling + another activity significantly reduced the probability of experiencing difficulties in maintaining PA during the health crisis. It is likely that individuals who cycled were more willing to replace other PA for more cycling and thus, felt less constrained. By contrast, the complementarity between activities was much more valued by participants who combined running (respectively, walking,) with other activities, so that they had the same probability of experiencing difficulties in their PA practice during the health crisis as their counterparts combining only indoor activities. It can be assumed that many individuals do not have enough disposition towards other activities as opposed to their initial choices to make substitutions during a period of disruption. This interpretation is again corroborated by the statistics from the National Barometer of Sports Practices which indicate that, in 2020, only 1 person out of 5 had the desire to practice a PA other than the one they usually practiced.

Consequently, while previous studies have shown that changes related to PA can be different for people who were highly active and less active/inactive before lockdown [32,33,35], the results of our study highlight that among exercisers, the nature of the physical activity(ies) practiced is paramount, and plays an even greater role than the frequency of the PA, on the sustainability of the practice. This result should be an important concern for policy makers: even among physically active people, solutions for varying the activities practiced must be created in order to avoid a serious temporary and potentially permanent populational decrease in PA, knowing that the substitution phenomena between activities seemed to be very limited during a health crisis. Reflections on how to optimize sports organizations and on the contents of the proposed sessions, as well as the use of digital tools, remains to be carried out in order to be better prepared in case of future restricted access to certain facilities, regardless of the reason.

The sedentary behavior of individuals pre-lockdown, as measured by screen time, was associated with greater difficulties in pursuing PA during lockdown, but only for the employed population. Certainly, all categories of the population (children, students, employed, retired; heavy or light digital users; physically active or inactive) have seen an increase in screen time since the beginning of the health crisis, with people becoming increasingly dependent on home Internet connections to telecommute, help children with schoolwork, or interact online with friends and family [24,39,41,42,43]. Overall, it is estimated that on average, digital device usage increased by 5h per day [44]. As for our employed population, 29% of these individuals spent between 10 and 14 h per day in front of a screen before lockdown; the lockdown increased the screen time up to 15–19 h per day, for those who were heavy users. Thus, this group is the most likely to experience excessive sedentary time, which is associated with an increased risk of depression [29,45], poorer mental health, and poorer physical health outcomes [46]. In particular, they are the most likely to suffer from the 3H syndrome, or a vicious circle of sedentary life [47]. A higher level of sedentary life (called hypokinesia), which is mechanically detrimental to PA (a day containing only 24 h), will lead to a loss of the aerobic capacity and the ability to make an effort to participate in PA (hypoxia). Any physical activity will thus cause a much greater and earlier fatigue than during a standard period, and the person’s ability to recover will also be impacted. As fatigue leads to displeasure in the practice of PA, the person will have great difficulty in performing usual PA. In the short term, the feeling of competence towards the activity will be impacted, which will lead the person to lose interest or to disengage from the activity, exhibited as hypodynamia, which is characterized by a decrease in strength and an increase in the barriers felt towards the practice of a PA. The person will then tend to decrease his or her investment in PA, reinforcing the initial hypokinesia phenomenon.

Conversely, among the employed population, the more sleep time the individuals exhibited before lockdown, the less difficulty they experienced in continuing PA during lockdown. Although some studies have shown that during the first lockdown, sleep debt was partially reduced due to telecommuting or short-time work implemented by many companies [48], it can be assumed that among pre-lockdown PA practitioners, those with high sleep time were more likely to pay attention to their lifestyle and thus, maintain their PA during the health crisis. In addition, because sleep time is an important recovery tool in sports practice [49], individuals who got more sleep had a greater ability to increase their PA time or to switch to new, possibly more physically demanding, activities.

In addition to low sleep time, being overweight or obese appeared to be a barrier to maintaining one’s PA during lockdown. Individuals with a high BMI prior to lockdown reported greater difficulty in maintaining PA. In the case of individuals with overweight or obesity, beliefs about the health benefits associated with PA, exercise self-efficacy, and physical and mental well-being are all strong contributors to the decision to engage in and maintain PA [50]. In addition, chronic joint pain, low self-esteem, as well as a restrictive or non-adapted lifestyle can have a negative impact on the practice of PA [51,52]. The constraints imposed by lockdown, and in particular, the closure of some sports establishments with specialized equipment and classes, as well as the cessation or lack of regularity of the group classes offered, may have played an important role in the sustainability of PA practice in this population by altering their motivation and reducing their opportunities to exercise. Moreover, even if the sports facilities were open, some people with overweight or obesity may have chosen not to attend crowded places, feeling that they could be more vulnerable to illness due to potential comorbidities [53].

Finally, few demographic characteristics were significant predictors of experiencing difficulties in continuing PA practice, except for sex in the employed population. Indeed, even with the same initial time dedicated to household chores, employed women experienced more difficulties in continuing PA than their male counterparts. This may be explained by the fact that during the lockdown, women were predominantly responsible for managing the schooling of their children at home, whether they were telecommuting or not [54,55], and had a greater increase in time spent doing household chores than men. Furthermore, as social interaction is one of the main reasons for women’s participation in PA [56], the closure of sports organizations such as gyms and associations may have negatively influenced their motivation. In this sense, the importance of the digital devices that were put in place as the pandemic progressed makes sense in helping to provide the continuity of daily PA practice. The non-significance of the sex variable in the regression performed on the total population, whereas a significant difference was apparent in the descriptive statistics, is essentially due to the fact that men and women do not practice the same type of activity. For example, men practiced less muscle-strengthening activities and more running, walking, or cycling than women [57].

In brief, several factors were identified by our study as predictors of experiencing difficulties in continuing PA during the COVID-19 crisis in France, including BMI, the type and number of physical activities usually practiced, along with the number of times per week dedicated to PA. For the employed population, specific factors were additionally decisive: sex, average screen time, and average nightly sleep time. Our study, however, has several limitations. First, our analyses were based on a specific population, since the survey was conducted among members of mutual insurance company groups. Although the subcategories of retired people and people with high disposable income were clearly overrepresented compared to the general French population, and the involvement of this group in prevention during the pandemic period made it possible to obtain a significant number of fully completed questionnaires (*n* = 460), our conclusions cannot be generalized to the broad French population due to our inclusion criteria. Second, participants’ difficulties in maintaining PA during the health crisis were self-reported and assessed via a simple yes or no question about problems posed by the closure of facilities. While our survey used a very common method of reporting activities [58], self-reported data has limitations. This method and the retrospective nature of the data collection excluded the possibility of objectively measuring PA levels (e.g., actimetry and TM6) before and after lockdown. Similarly, basing our analysis on a comparison of self-reported before/after measures would likely have been unreliable. We believe that the retained type of simple question focusing on the “lockdown shock impact” on usual practices generates more reliable answers considering a self-report evaluation. We are aware, however, that there may be barriers other than just facility closures that have made it difficult to participate in PA, such as changes in work design or caregiving responsibilities. Particularly, in-depth studies of these complementary difficulties would be important to distinguish the differing effects on men and women, given the gender differences already observed in our employed population.

## 5. Conclusions

The global spread of COVID-19 had a strong impact on exercise patterns all over the world. While people were encouraged to exercise non-intensively during the lockdown to stimulate their mental and physical health, the French lockdown heavily reduced people’s range of opportunities to exercise. This simultaneous combination of exercise promotion and restriction seems to have heterogeneous effects, depending on the individual pre-pandemic exercise-related characteristics, and in particular, on the nature of the physical activity(ies) usually practiced.

Our results contribute to increasing the abilities of public health policy makers and stakeholders in PA and prevention to better target the populations with the greatest difficulty in maintaining PA (among regular practitioners outside of a disruption period such as that of COVID-19) without having to measure an actual decrease in PA. Special attention should then be paid to how to encourage people who spend a lot of time daily on screens, get little sleep, and/or have a high BMI, to exercise during lockdown. As these groups are often cited as having the most difficulty maintaining their PA over the long term, outside of a health crisis, any solution developed to encourage their PA practice during a lockdown could prove effective during any period of disruption in their habits, if not in general.

Now that it is known that a large percentage of individuals encountered difficulties in continuing PA during the health crisis, future scholarship is encouraged to look into potential long-term effects of this disruption in PA by means of longitudinal study designs and objective measures of individual PA duration, frequency, and intensity before, during, and after the lockdown (or other COVID-19-related restrictions). In particular, such complementary studies will shed light on whether the changes in PA levels risk not being sustainable, as predicted by the self-determination theory, since they are rooted in extrinsic motivations (i.e., lockdown regulations and restrictions). In this perspective, the development of a survey on intrinsic motivations and the psychological dimension in order to identify the influence of certain behavioral biases would be more than relevant.

## Figures and Tables

**Table 1:** Descriptive statistics of the study population.

	Total Sample (*n* = 460)								Subsample in Employment (*n* = 238)			
			Experiencing Difficulties in Practicing PA during the Health Crisis?						Experiencing Difficulties in Practicing PA during the Health Crisis?			
	All (*n* = 460)		Yes (*n* = 196)		No (*n* = 264)		All Employed (*n* = 238)		Yes (*n* = 101)		No (*n* = 137)	
	*n*	%, Mean	*n*	%, Mean	*n*	%, Mean	*n*	%, Mean	*n*	%, Mean	*n*	%, Mean
Individual characteristics:												
Sex:												
Men	313	31.96%	80	40.82%	65	25.38% ^+++^	59	24.79%	35	34.65%	24	17.52% ^+++^
Women	147	68.04%	116	59.18%	197	74.62% ^+++^	179	75.21%	66	65.35%	113	82.48% ^+++^
Average age (years)		57.47 (12.44)		57.56 (12.66)		57.41 (12.30)		48.68 (10.20)		48.56 (10.50)		48.76 (10.02)
Lives:												
Alone	146	31.74%	59	30.10%	87	32.95%	59	24.79%	21	20.79%	38	27.74%
In a couple	268	58.26%	115	58.67%	153	57.95%	144	60.50%	63	62.38%	81	59.12%
With other people	46	10.00%	22	11.23%	24	9.10%	35	14.71%	17	16.83%	18	13.14%
Annual taxable household income (EUR):												
<20,000	37	8.04%	21	10.71%	16	6.06%	13	5.46%	6	5.94%	7	5.11%
(20,000–30,000)	109	23.70%	51	26.02%	58	21.97%	51	21.43%	23	22.77%	28	20.44%
(30,000–50,000)	147	31.96%	60	30.61%	87	32.95%	74	31.09%	29	28.71%	45	32.85%
50,000 or more	93	20.22%	39	19.90%	54	20.45%	67	28.15%	31	30.69%	36	26.28%
Not declared	74	16.09%	25	12.76%	49	18.56%	33	13.87%	12	11.88%	21	15.33%
Occupational status:												
Civils servants	71	15.43%	27	13.78%	44	16.67%	71	29.83%		26.73%		32.12%
Managers/executives	91	19.78%	37	18.88%	54	20.45%	91	38.24%		36.63%		39.42%
Technicians, Clerks	19	4.13%	8	4.08%	11	4.17%	19	7.98%		7.92%		8.03%
Service workers	46	10.0%	24	12.24%	22	8.33%	46	19.33%		23.76%		16.06%
Other occupations	15	3.27%	6	3.06%	9	3.41%	11	4.62%		4.95%		4.38%
Retired	218	47.39%	94	47.96%	124	46.97%		---		---		---
Health, PA and sedentary behaviors before lockdown:												
Body Mass Index		23.76 (5.03)		23.62 (4.39)		23.87 (5.47)		23.63 (5.10)		23.46 (4.69)		23.75 (5.40)
Average number of PA sessions per week		3.94 (1.86)		4.33 (1.96)		3.65 *** (1.73)		3.59 (1.84)		3.88 (1.89)		3.38 ** (1.78)
Average time spent in PA per week (hours)		5.06 (3.21)		5.48 (3.50)		4.73 ** (2.95)		4.37 (2.77)		4.65 (2.90)		4.18 (2.67)
Number of different activities usually practiced		2.33 (1.12)		2.12 (1.08)		2.50 *** (1.13)		2.39 (1.17)		2.25 (1.20)		2.50 * (1.15)
Average daily screen time (hours)		5.33 (3.32)		5.11 (3.34)		5.48 (3.31)		7.41 (2.95)		6.95 (2.97)		7.74 ** (2.90)
Average sleep time per night (hours)		7.15 (1.00)		7.18 (0.96)		7.12 (1.04)		7.13 (0.93)		7.20 (0.91)		7.08 (0.94)
Average weekly housework time (hours)		5.52 (3.83)		5.17 (3.53)		5.78 * (4.02)		4.93 (3.22)		4.51 (2.92)		5.24 * (3.39)

Notes: standard deviation in parenthesis; * *p* < 0.10, ** *p* < 0.05, *** *p* < 0.01, with *p* as the *p*-values associated to mean-comparison t-tests indicating significant differences between participants with and without difficulties in continuing their usual physical activity; ^+++^ *p* < 0.01, with *p* as the *p*-values associated to exact chi-square tests.

**Table 2:** The results of the multivariate logistic regressions explaining difficulties in practicing PA during the health crisis.

	Total Sample		Subsample in Employment	
	aOR	|z|	aOR	|z|
Individual characteristics:				
Sex:				
Men	0.708	(1.25)	0.398 **	(2.21)
Women	Ref.	Ref.	Ref.	Ref.
Age	0.998	(0.15)	1.004	(0.22)
Lives:				
Alone	1.381	(0.75)	1.918	(1.16)
In a couple	0.738	(0.75)	0.771	(0.51)
With other people	Ref.	Ref.	Ref.	Ref.
Annual taxable household income (EUR):				
<20,000	Ref.	Ref.	Ref.	Ref.
(20,000–30,000)	1.916	(1.40)	0.711	(0.41)
(30,000–50,000)	3.147 **	(2.37)	1.728	(0.63)
50,000 or more	2.364	(1.57)	1.250	(0.24)
Not declared	4.665 ***	(2.83)	2.996	(1.17)
Occupational status:				
Civil servants	0.674	(0.94)	Ref.	Ref.
Managers/executives	0.431 *	(1.68)	0.678	(0.77)
Technicians, Clerks	0.379	(1.52)	0.680	(0.59)
Service workers	0.401 *	(1.72)	0.736	(0.61)
Other occupations	0.505	(1.07)	0.536	(0.85)
Retired	Ref.	Ref.	---	---
Health, PA and sedentary behaviors prior to the lockdown:				
BMI	1.059 **	(2.15)	1.062 *	(1.66)
Number of PA sessions per week	0.758 ***	(3.44)	0.756 **	(2.11)
Average time spent in PA per week	0.985	(0.34)	1.043	(0.51)
Number of different activities usually practiced	1.637 ***	(2.65)	1.581 *	(1.74)
Average daily screen time	1.061	(1.30)	1.121 *	(1.79)
Average sleep time per night	0.893	(0.95)	0.706 *	(1.83)
Average weekly housework time	1.024	(0.74)	1.050	(0.93)
PA:				
Only cycling/homebike	0.044 ***	(3.58)	0.174 *	(1.71)
Only walking/Nordic walking	0.145 ***	(4.22)	0.076 ***	(3.43)
Only running	0.108 ***	(2.94)	0.040 ***	(2.78)
Only muscle-strenthening/weight training	1.525	(0.56)	1.710	(0.58)
Running + walking	0.040 ***	(2.74)	---	---
Walking + cycling	0.091 ***	(4.31)	0.063 ***	(2.76)
Other combination with walking	0.683	(1.20)	0.838	(0.41)
Other combination with running	0.630	(1.41)	0.723	(0.71)
Other combination with cycling	0.427 ***	(2.72)	0.289 ***	(2.83)
Other sports/combinations	Ref.	Ref.	Ref.	Ref.
Nb of observations	460		238	

Note: * *p* < 0.10, ** *p* < 0.05, *** *p* < 0.01; aOR: adjusted odds ratio, z-statistics in parenthesis; Ref.: reference category for the categorical data.

## Data Availability

The data presented in this study is available on request from the corresponding author.
